# An On-Time Power-Aware Scheduling Scheme for Medical Sensor SoC-Based WBAN Systems

**DOI:** 10.3390/s130100375

**Published:** 2012-12-27

**Authors:** Tae-Ho Hwang, Dong-Sun Kim, Jung-Guk Kim

**Affiliations:** 1 Multimedia IP Center, Korea Electronic Technology Institute, 6th Fl., #22, Daewangpangyo-ro 712 Bundang-gu Gyeonggi-do, Seongnam-si 463-400, Korea; E-Mail: dskim@keti.re.kr; 2 Department of Computer Engineering, Hankuk University of Foreign Studies, Gyeonggi-do, Yongin-si 449-791, Korea; E-Mail: jgkim@hufs.ac.kr

**Keywords:** on-time scheduling, power-aware, WBAN, wakeup-radio, wakeup-timer, sensor SoC, ICD, TMO

## Abstract

The focus of many leading technologies in the field of medical sensor systems is on low power consumption and robust data transmission. For example, the implantable cardioverter-defibrillator (ICD), which is used to maintain the heart in a healthy state, requires a reliable wireless communication scheme with an extremely low duty-cycle, high bit rate, and energy-efficient media access protocols. Because such devices must be sustained for over 5 years without access to battery replacement, they must be designed to have extremely low power consumption in sleep mode. Here, an on-time, energy-efficient scheduling scheme is proposed that performs power adjustments to minimize the sleep-mode current. The novelty of this scheduler is that it increases the determinacy of power adjustment and the predictability of scheduling by employing non-pre-emptible dual priority scheduling. This predictable scheduling also guarantees the punctuality of important periodic tasks based on their serialization, by using their worst case execution time) and the power consumption optimization. The scheduler was embedded into a system on chip (SoC) developed to support the wireless body area network—a wakeup-radio and wakeup-timer for implantable medical devices. This scheduling system is validated by the experimental results of its performance when used with life-time extensions of ICD devices.

## 1. Introduction

Owing to the increase in today's aging population there has been a gradually increasing demand for medical implantable sensor devices such as pacemakers and defibrillators. A major issue in this field has been the development of technologies for wireless networking between implantable devices [1–3]. One major goal of using this type of communication is to be able to externally monitor patients' health and the status of the devices they wear, both internally and externally. IEEE 802.15.6 [4] is a wireless body area network (WBAN) standard for communications between medical implantable devices [5]. It specifies a frequency band and a Medium Access Control (MAC) protocol as its Physical Layer (PHY) for in-body and on-body applications.

Since implanted medical devices are designed so that they will not need to be accessed for maintenance over a long period of time, high-level constraints on power consumption are needed that can sustain a device over several years with a duty-cycle of under 1%. Thus, the design of an extremely low power RF transceiver [6,7] and a low-power system on chip (SoC) has been an important issue.

In addition, approaches that utilize a wakeup-radio channel [8,9] and an energy-efficient protocol [10–12] to minimise the sleep mode current have been introduced. For devices that require a low duty-cycle and a long lifetime, power management in sleep mode is far more important than it is in active mode. For this reason, the use of a wakeup-radio channel has been included in the IEEE 802.15.6 WBAN standard as an option. Each hardware block of a sensor-based WBAN device is woken up from the power-off state by an external wakeup radio or by the schedule of a device. Therefore, a device that uses an external wakeup radio and timer must be able to support efficient saving and restoration of the contexts of communication, and sensors that transmit to or from non-volatile memory. In addition, the power states of each hardware block, such as a modem, and RF and I/O peripherals, must be managed on the system level, so that the system power spectrum is reconfigured along with its forward task scheduling. Although low-power operations are important for sensor devices, operational reliability must be sustained prior to power consumption in the case of medical applications. Applications for medical sensor devices must guarantee the on-time execution of periodic sensor data processing and actuator control. For this reason, sensor device applications are usually implemented based on a power-aware scheduler that can manage both on-time periodic activities and the power consumption of tasks.

There are two typical approaches to scheduling for medical sensor devices. One is the event-driven scheduling approach [13]. Although it is simple and provides a predictable single task model, there can be delays in data processing because it processes events in first-in first-out (FIFO) fashion with a single task. The other approach is the pre-emptive multi-thread scheduling scheme, which is generally used in popular operating systems such as μCOSII and MANTIS [14]. While it offers the benefit of ease of design of complex applications, it has difficulty in supporting on-time predictable scheduling, owing to pre-emptions and resource conflicts between tasks. Without predictable scheduling, a system cannot enter a deep sleep mode. This paper presents a new scheduling scheme that encompasses the benefits of both approaches, as it minimizes power consumption and guarantees on-time task execution by increasing the predictability of a system, while using multi-thread scheduling.

Typical applications for a sensor node can be organized to include time-triggered periodic tasks and event-triggered sporadic tasks. If a system can be organised only to manage periodic tasks, the system's predictability can be assured, and it becomes quite easy to determine the time to enter a deep sleep mode. For systems in which event-triggered sporadic tasks cannot be avoided, two approaches to increase the predictability of a system have been introduced. One of these is the time-triggered message-triggered object (TMO) scheme [15,16]. In the TMO scheme, all executions of periodic tasks are scheduled with pre-emption in a deadline-based manner. However, the execution of each sporadic task is non-pre-emptible, and it can be postponed if there is a potential overlap of executions between the nearest periodic task and a sporadic task in the future. This TMO scheduling scheme, which is called basic concurrency constraint (BCC) [16], increases the predictability when sporadic tasks must be managed. The other approach is a modified form of dual-priority scheduling (DPS) [17,18], which resolves the conflicts between periodic and sporadic tasks by giving higher priority to periodic tasks, while allowing pre-emptions. In this system, the speed of a CPU is determined based on the deadlines and the worst case execution times (WCETs) of the tasks, thus offering improved power management. In both approaches, however, on-time scheduling and the completion of a periodic task are not guaranteed, because of pre-emptive deadline-based scheduling, so planned static power adjustment based on the predictive scheduling of periodic tasks is impossible.

The scheduler presented in this paper represents a new approach, which supports a planned power management scheme by using predictive scheduling based on the non-pre-emptive serialization of periodic tasks, while incorporating the concepts of TMO's BCC and the DPS. At the design stage of a task system, all periodic tasks are serialized first by adjusting their initial offsets so that there is no resource conflict, no pre-emption, and no execution overlap, in order to guarantee the on-time invocation and completion of tasks. Once periodic tasks are serialized by a tool such as the one described in [19], deadline-driven scheduling is not necessary, so it becomes possible to determine whether or not to postpone the scheduling of a sporadic task. That is, the scheduling of a sporadic task is postponed when the system predicts a potential overlap between the execution of a sporadic task and the nearest periodic task. This prediction allows for a more planned and efficient power management. To implement this scheduling strategy, four major power modes were defined and applied to the scheduler so that each power mode supports the minimum consumption for each possible scheduling scenario. In particular, the scheduler is designed to maximize the duration of the deep sleep mode, based on its predictive scheduling.

This paper is organized as follows: Section 2 describes the design of an on-time power-aware scheduler that is a realization of the above ideas, based on a dual priority scheduler. In Section 3, the hardware structure of the developed chipset and the WBAN PHY/MAC protocol on an SoC are briefly presented. Finally, in Section 4, we present discussion of the experimental results, and estimations based on a simulation of the scheduler and its applicability to implantable devices.

## 2. On-Time Power-Aware Scheduler

### 2.1. System, Task, and Power-Mode Model

In the developed system, a sensor-node application consists of several non-pre-emptible atomic tasks. Tasks are divided into periodic tasks and sporadic tasks. All tasks are scheduled only when the system is in an ACTIVE state. When the system is put into an INACTIVE state, by either the application or the scheduler, scheduling is prohibited until the system is made ACTIVE by either a wakeup-radio signal or a wakeup-timer. In addition to the task and system model, the power mode model and the operation model of peripheral devices such as sensors and actuators must be considered. The following are descriptions of the task, device, and power-mode models.

**Notation 1**: A periodic task and its nth periodic execution are denoted by *TP_i_* (*o*, *p*, *w*, *g*, *m*) and ${\mathrm{TP}}_{i}^{n}$, respectively.

where

*i* is the identifier of a periodic task,
*o* is the initial offset of a periodic task,
*p* is the period of a periodic task,
*w* is the WCET of a periodic task,
*g* is the guard time of a periodic task, and
*m* is the power mode requested to run a periodic task.

The guard time of a periodic task is the predefined start-up time needed to enable the power and hardware devices for running. All executions of periodic tasks in the system's ACTIVE mode can be serialized by a schedulability analysis tool [19] in order to prevent the pre-emption and overlap of executions. Thus, the scheduler can schedule periodic tasks in an on-time FIFO manner and can predict the timing characteristics in regard to their execution. The states of periodic tasks are classified into RUNNABLE and INACTIVE. The state of a periodic task becomes RUNNABLE when the *time_left* value in its task control block (TCB) falls into a non-positive value because the *time_left* value is the amount of time left until the next periodic invocation. Upon the completion of a periodic job, a periodic task becomes INACTIVE.

**Notation 2**: A sporadic task and its nth execution are denoted by *SP_i_* (*e*, *w*, *m*) and ${\mathrm{SP}}_{i}^{n}$, respectively.

where

*i* is the identifier of a sporadic task,
*e* is the event that triggers a sporadic task,
*w* is the WCET of a periodic task, and
*m* is the power mode requested to run a sporadic task.

A periodic task can directly activate a sporadic task by sending an event to a sporadic task. In addition, a sporadic task can be activated by a device that was initialized by a periodic task. All devices of the system, such as sensors and actuators, conform to a typical activity sequence, as follows. Each device must be initialized by a periodic task in order to function. After initialization, the device works independently and then generates an interrupt. The interrupt handler again generates an event to trigger the sporadic task that is responsible for post-processing. For example, a sensor device completes the sampling of data following its initialization by a periodic task, and it then signals the end of sampling by generating an interrupt. The sporadic task that is triggered by the interrupt sends the sensor data to an external device via a UART port, when it is scheduled.

Unlike the scheduling of periodic tasks, the scheduling of an activated sporadic task is postponed when an overlap is predicted between the execution of the sporadic task and the execution of the nearest periodic task. The states of sporadic tasks are classified as SUSPENDED, INACTIVE, or RUNNABLE. When a sporadic task terminates its execution and there is no initialized event source for the sporadic task, the state is set to SUSPENDED. Once the event-source device is initialized by another periodic task, the sporadic task waits for the event in an INACTIVE state. When the awaited event eventually occurs, the sporadic task goes into a RUNNABLE state. However, a RUNNABLE sporadic task can only be scheduled immediately when the following three conditions are met:

- There is no current execution of another task.
- There is no preceding RUNNABLE sporadic task.
- It is predicted that the execution of the sporadic task will not disturb the future execution of the coming periodic task, considering the WCET of the sporadic task.

**Figure 1.**
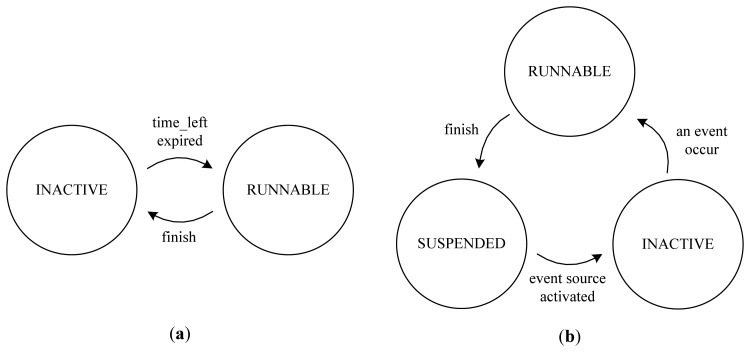
(**a**) State transition of a periodic task. (**b**) State transition of a sporadic task.

If these conditions are not met, the sporadic task must wait to be scheduled until the three conditions are met in the RUNNABLE state. Figure 1 presents a diagram of the transition of task states with the scheduler.

With the above task model, four power modes are supported for power-aware scheduling: task-power mode, *wait_event*, *sleep_with_timer*, and *sleep_with_radio*. The *sleep_with_radio* and *sleep_with_timer* are modes for the system's INACTIVE state, and the others are for its ACTIVE state. The task-power mode is a task-specific mode, in that only necessary devices are turned on for the execution of a task. This power mode also includes the configurations of the microcontroller unit (MCU) and memory. When no task is being run, the system's power mode can be any one of the following: *wait_event*, *sleep_with_timer*, or *sleep_with_radio*. In the *wait_event* mode, the MCU and the system timer are enabled with the lowest possible power support and the lowest possible clock frequency, such as 32 kHz oscillator. Devices such as sensors and transceivers that are enabled by a periodic task can be kept enabled in this mode following the termination of a periodic task. Therefore, the *wait_event* mode indicates a power mode, in that a periodic task or a sporadic task can be scheduled in the near future. The *sleep_with_timer* mode is a lower-power mode, in that all system power supports are turned off, except for the wakeup timer. The system enters into *sleep_with_timer* when there is no RUNNABLE sporadic task, and when the amount of the time left until the next invocation of a periodic task is greater than *minSleepInterval*. The predefined value of *minSleepInterval* is the amount of time that is required for the system to recover its power in order to execute a task. Finally, the system enters into *sleep_with_radio* upon the request of an application. The *sleep_with_radio* is the lowest-power mode, in that all system power supports are turned off except for the wakeup-radio receiver. The contexts of the registers and memory must be saved in non-volatile memory when the system enters into either the *sleep_with_timer* or the *sleep_with_radio* mode. Additionally, the saved contexts must be restored when the system exits from one of these modes.

A power mode transition is performed by the scheduler calling the *Power_Adjust()* function. The *Power_Adjust()* function is designed to reconfigure hardware according to a given power descriptor before and after a task receives a schedule. The reconfiguration of hardware includes the modification of the MCU clock rate, voltage scaling, turning on of the peripherals, and scaling of the system timer.

### 2.2. Design of an On-Time Power-Aware Scheduler

**Figure 2.**
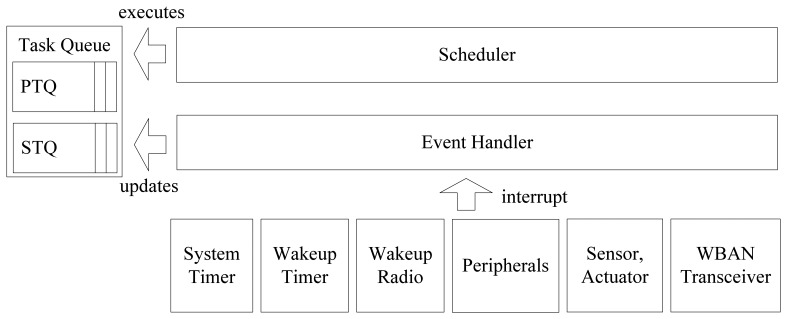
Structure of on-time power-aware scheduling system.

The scheduling system consists of two main modules, the task scheduler and the event handler. The scheduler keeps the TCBs of tasks either in the periodic task queue (PTQ) or in the sporadic task queue (STQ), as shown in Figure 2.

The event handler is responsible for handling hardware interrupts from the system timer, wakeup timer, wakeup radio, peripherals, and the WBAN transceiver. For example, when an interrupt from the system timer occurs, it updates the time-left values of all periodic tasks. If a task with a non-positive *time_left* is found, the state of the task is changed from INACTIVE to RUNNABLE, and the *time_left* value is reloaded with the value of that period, minus *guard_time*, to allow the system to prepare the start-up for the task. When there is an interrupt from a device, the handler prepares an event for the sporadic task that is responsible for that device and changes the state of the sporadic task from INACTIVE to RUNNABLE. If the event handler receives either a wakeup-timer interrupt or a wakeup-radio interrupt, it resumes the operation of the system by restoring the system context. The scheduler is called when the handling of the interrupt is complete.

In addition to handling hardware interrupts, the event handler also generates an event to trigger a sporadic task at the request of a periodic task, and as a result, the event handler changes the state of the target sporadic task from INACTIVE to RUNNABLE. In this case, the scheduler is not called immediately, because the periodic task that requested the event is still running.

**Figure 3.**
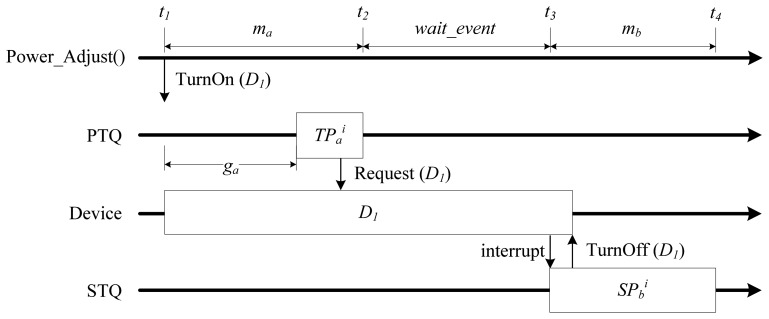
Typical power mode transition scenario.

**Figure 4.**
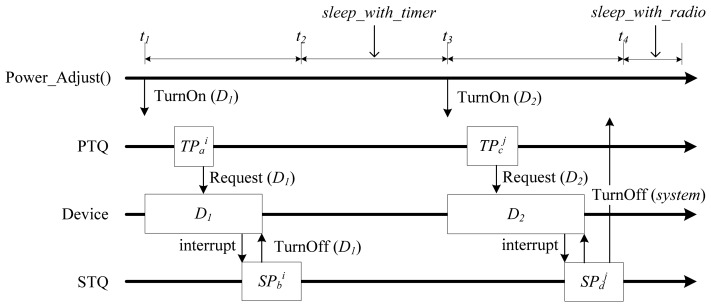
Power mode transition scenario using *sleep_with_timer* and *sleep_with_radio*.

**Figure 5.**
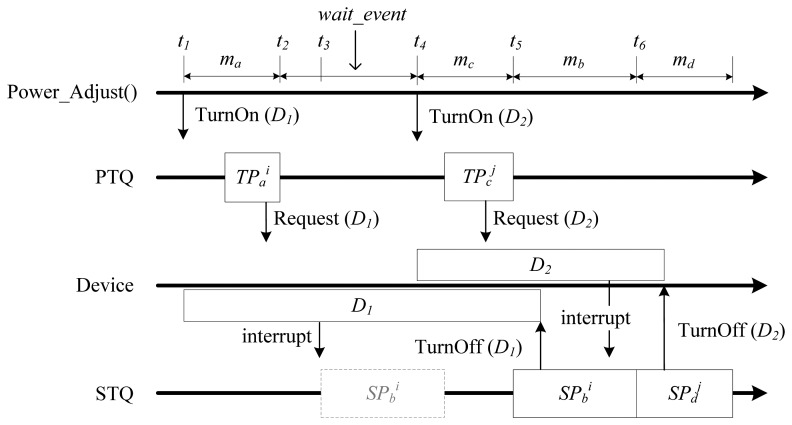
Power mode transition scenario when there is an overlap of executions.

Figures 3, 4 and 5 graphically illustrate several scheduling and power adjustment scenarios. In Figure 3, *m_a_* and *g_a_* denote the power mode and guard time, respectively, of the task *a*. In the beginning, the scheduler sets the power mode as *m_a_* for *TP_a_* and initializes the device *D_1_* at the time of *t_1_*, which is prior to the starting point of the task instance *TP_a_^i^* owing to its *g_a_*. The execution of the periodic task instance *TP_a_^i^* activates the sensor device *D_1_* by calling *Request()*. The system then changes the state of the sporadic task *SP_b_* from the SUSPENDED state to INACTIVE in order to allow the task to wait for an event. When *TP_a_^i^* terminates at *t_2_*, the scheduler turns the system power mode to *wait_event*, because there is an INACTIVE sporadic task whose execution is potentially possible in the near future. When an interrupt from *D_1_* occurs at *t_3_*, the system determines whether or not to schedule the sporadic task *SP_b_* by considering the WCET of *SP_b_*, as well as the earliest possible start time of the upcoming periodic task. In Figure 3, when *SP_b_* is scheduled, the power mode is set to the task-power mode *m_b_*, since no execution will overlap with a periodic task, and *SP_b_* then terminates.

When *SP_b_* is completed at *t_2_* in Figure 4, the task state is set to SUSPENDED again. At this time, the power mode is determined to be either *wait_event* or *sleep_with_timer* by considering the time left until the next periodic task and the number of sporadic tasks that are INACTIVE. At *t_2_*, the power mode is set to *sleep_with_timer* because there is no INACTIVE sporadic task, and the time left until the next periodic task is longer than *minSleepInterval*. At *t_4_*, if *SP_d_* calls *TurnOff(system)*, the system enters into the *sleep_with_radio* mode.

In Figure 5, when an interrupt from *D_1_* occurs at *t_3_*, the scheduler knows that *SP_b_^i^* overlaps with *TP_c_^j^*, the execution of the next periodic task, by comparing the WCET of *SP_b_* with the nearest time-left value. As a result, scheduling of the sporadic task *SP_b_^i^* is postponed.

Overall, the scheduler assumes control when the event handler completes a cycle of interrupt processing and when the running of a task is complete. The scheduler action is straightforward, since it considers the task model, power mode model, and the time when the scheduler takes control of the CPU, as follows:

1. If there is at least one RUNNABLE periodic task, set the task-power mode and call the task.
2. Otherwise, if there is at least one RUNNABLE sporadic task, determine whether or not it can be scheduled immediately by comparing the WCET of this task and the *time_left* value of the next periodic task. If immediate scheduling is possible, set task-power mode and call the task. If it is not possible, set the system power mode as *wait_event*.
3. If there is no RUNNABLE task but there is at least one INACTIVE sporadic task, set the power mode as *wait_event*.
4. If there is no RUNNABLE or INACTIVE task,
  4.1 If there was a *turn_off_system* request from a task, set the power mode as *sleep_with_radio*.
  4.2 Else if the time left until the invocation of the next coming periodic task is larger than *minSleepInterval*, set the power mode as *sleep_with_timer*. Otherwise, set *wait_event* mode.

**Figure 6.**
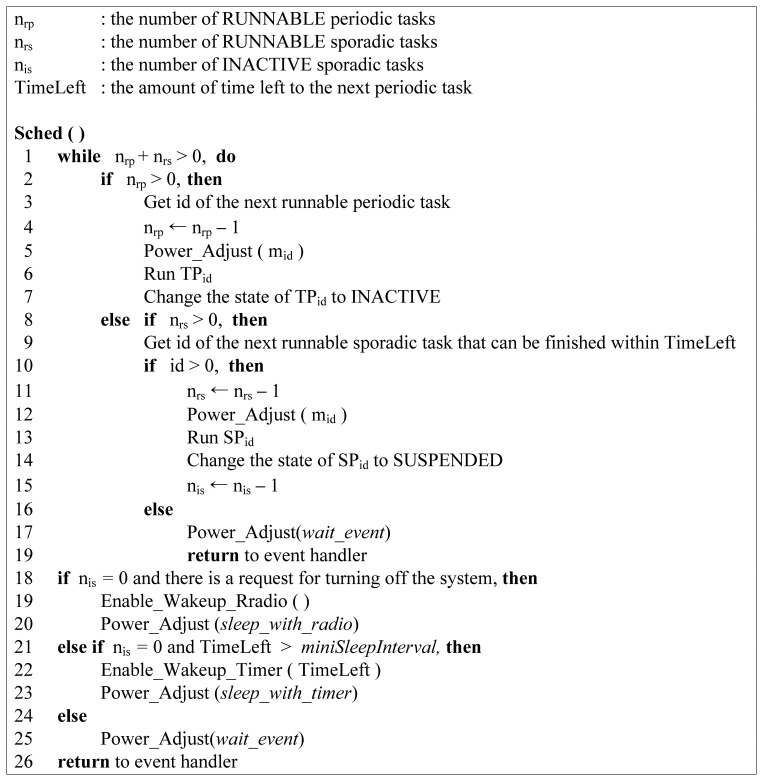
Operations of on-time power-aware scheduler.

As given above, the running of a task is implemented in the form of a call to a task-body by the scheduler, because all tasks run without pre-emption. Figure 6 outlines the scheduler's actions in detail.

## 3. Implementation

**Figure 7.**
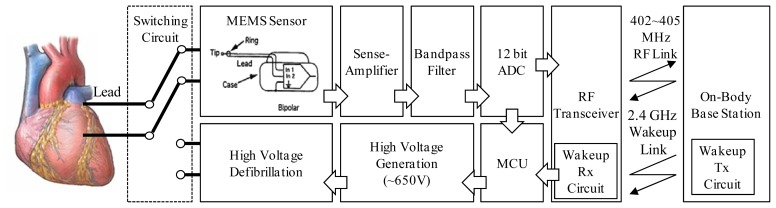
Structure of the ICD device.

The proposed scheduler was deployed for use with an implantable cardioverter-defibrillator (ICD) device that was developed in a project called “Component Development of Ultra-Low Power ICTS”, which was supported by the Korean government. The ICD is a small battery-powered electrical impulse generator that is implanted into a patient who is at risk of sudden cardiac death due to ventricular fibrillation and ventricular tachycardia. The device is programmed to detect cardiac arrhythmia and correct it by delivering a jolt of electricity. The device can also transmit health status information and alarms to an on-body device. The structure of the ICD device is shown in Figure 7. A problem in a patient's heart can be detected by the difference between the voltages of the two ICD leads that are inserted into the heart. The analogue signal is amplified and then converted into a digital signal by means of 12-bit analogue-to-digital (ADC) after noise filtering. The acquired electrocardiography (ECG)/electromyography (EMG) data are transmitted to an external monitoring device and are processed by the application of a cardiac arrhythmia detection device.

**Figure 8.**
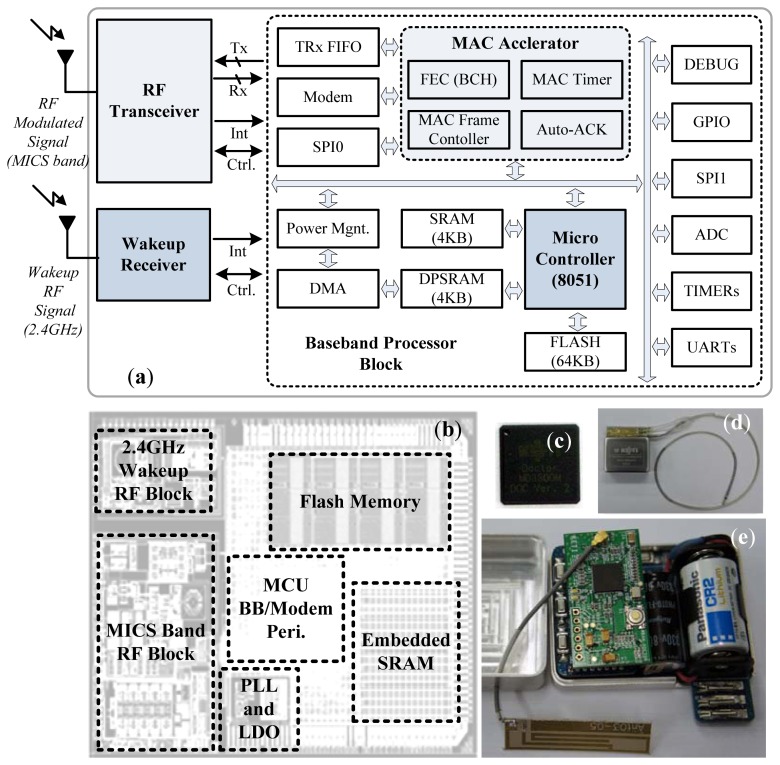
Hardware platform with an SoC for implanted medical devices. (**a**) Internal block diagram of the SoC. (**b**) SoC layout. (**c**) WBAN SoC. (**d**) Bipolar lead connection to the implant defibrillation device (medical Ti case, 56 × 43 × 22 mm). (**e**) Modules for arrhythmia detection/defibrillation (ECG/EMG signal analyser/650 V stimulation), and the transceiver SoC module.

For the ICD device, an SoC and its hardware platform were implemented, as shown in Figure 8. The transceiver module is responsible for transmitting ECG/EMG signals and signalling an arrhythmia by means of an alarm.

The developed SoC consists mainly of a digital modem, an 8-bit micro-controller, and MAC hardware. The digital modem has a modulator with a 6-bit digital-to-analogue converter (DAC) and a demodulator with a 4-bit ADC. The 24/12/6-MHz micro-controller executes the 8051 instruction set and supports 64 kB of program memory for the software MAC protocol and applications. It also provides a four-channel direct memory access (DMA) controller that is used in the saving and restoration of contexts.

The micro-controller also supports four hardware interrupts for two full-duplex serial communication interfaces and two 16-bit timers. The hardware MAC supports cyclic redundancy check (CRC), forward error correction (FEC), and four programmable timers in order to check the timing rules of the MAC. It also supports the 128-bit Advanced Encryption Standard (AES). Table 1 briefly lists the PHY characteristics.

**Figure 9.**
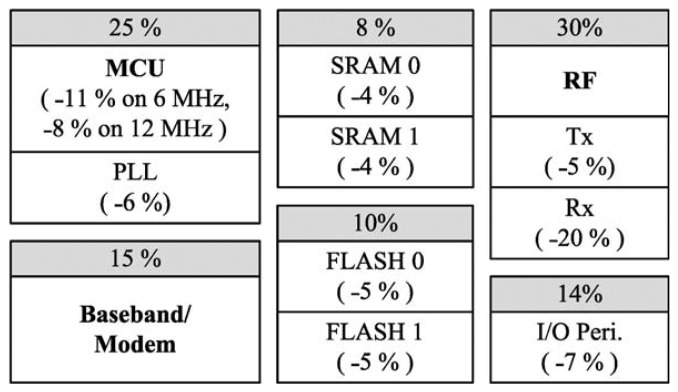
Power consumption and saving ratios of power-controllable hardware components.

Figure 9 presents a profile of the power controllable hardware components. In the figure, the power consumption ratio of each block is represented as a percentage. Each minus value enclosed in parentheses represents the power saving ratio when a block is turned off. The scheduler turns each component on and off using the Power Management Unit (PMU) according to the power descriptor of a given task. Two requirements of the developed ICD are that it must be sustained over several years with a low duty-cycle (avg. 0.3–0.6%) and must support a high bit rate (about 300 kbps) communication. By considering an environment with a high packet error rate [20], it employs the selective automatic repeat request (ARQ) [21] and the Bose, Chaudhri, Hocquenghem (BCH) block code for FEC. All necessary functions of the protocol were implemented with a number of periodic and sporadic tasks. The application to detect a cardiac arrhythmia was also organized with one sporadic and one periodic task. Once an in-body device has been alerted by a 2.4 GHz radio signal, it makes a handshake with a monitoring device and then begins sensing and transmitting data.

## 4. Experimental Results

**Figure 10.**
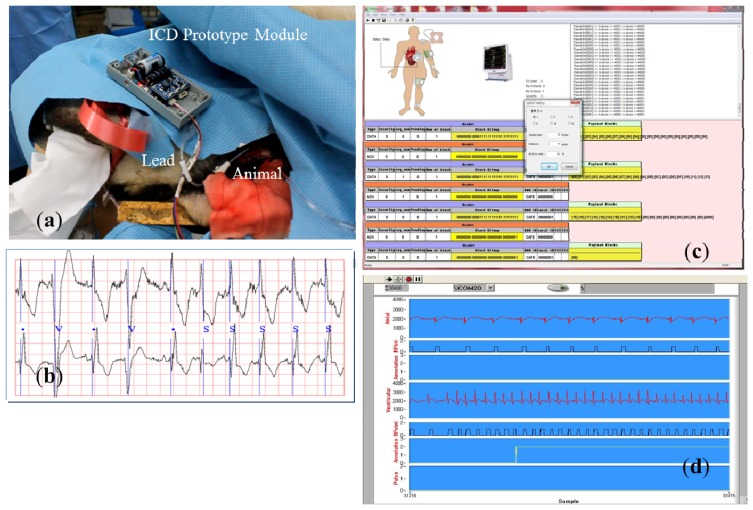
(**a**) Experiment with an animal. (**b**) Detection of ECG/EMG signal. (**c**) MAC frame sniffing. (**d**) Remote monitoring of ECG/EMG signals.

Several experiments with an animal were performed in order to verify the operation of the ICD and the scheduler. Figure 10 shows the main steps of the experiments.

**Figure 11.**
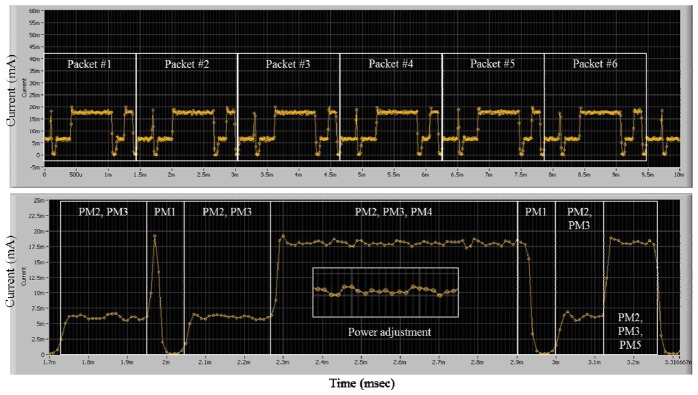
Amounts of power consumed by on-time power-aware scheduler during transmission of packets.

In order to measure the basic power consumption of the SoC's communication module, a National Instrument PXI 4071 multi-meter device was used, with a transmit data length of 20 bytes, a supply voltage of from 1.8 V to 3.3 V, and an MCU clock rate of zero or from 32 K to 32 MHz. The graph in the upper part of Figure 11 shows the amounts of power consumed according to the operation time of continuous data transmissions. The graph in the lower part of the figure shows the amounts of power consumed according to the power mode in a packet transmission.

Table 2 provides descriptions of the six power modes and the average amount of current consumed in each mode. A task that accesses the transceiver works at either PM4 or at PM5. A task that performs sensing via the ADC runs in the PM3 mode. In the duration of waiting for an event, the scheduler changes the power mode to the PM2 mode. After the processing is complete, the system enters into PM1 or PM0 in order to sleep.

**Figure 12.**
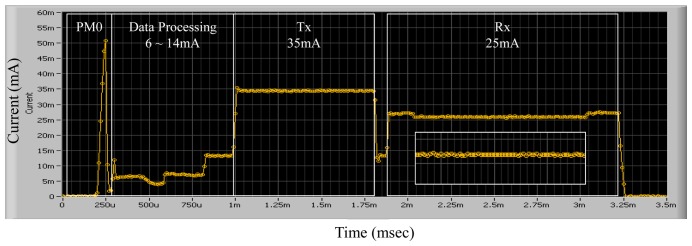
Measurement of current consumed by μCOSII-based system during transmission of a packet.

The developed system has been compared with a μCOSII-based system. A μCOSII-based system [22] with a normal multi-tasking scheduler, however, only supports a simple form of power management, in that only the *sleep()* function and *sleep_with_radio* are supported. That is, the μCOSII-based system does not use the *Power_Adjust()*, the *wait_event*, or the *sleep_with_timer* modes. As can be seen in Figure 12, the amount of power consumed by the μCOSII-based system increased because no power adjustment and no scaling of the MCU voltage/clock were used for a task. In addition, the operation time of the hardware was extended, because the related hardware must be previously turned on for the on-time execution of the protocol.

Based on measurements of the basic power consumption, a simulation-based estimation of the lifetimes of the two systems was performed. The power consumption of a device can be defined as the sum of the amounts of current consumed in each power mode [23]. These amounts can be obtained by multiplying the necessary current *I* of a given mode by the work time *t*, where *Q_total_* is amount of electric charge consumed by the module, as follows:

(1) $$Q_{\text{total}}=\sum_{i=PM\;0}^{PM5}t_{i}\cdot I_{i}$$

The lifetime of an ICD device can be estimated by using the amount of current it consumes, where *Q_battery_* is the capacity of battery:

(2) $$\text{Lifetime}=\frac{Q_{\text{battery}}}{Q_{\text{total}}}$$

**Figure 13.**
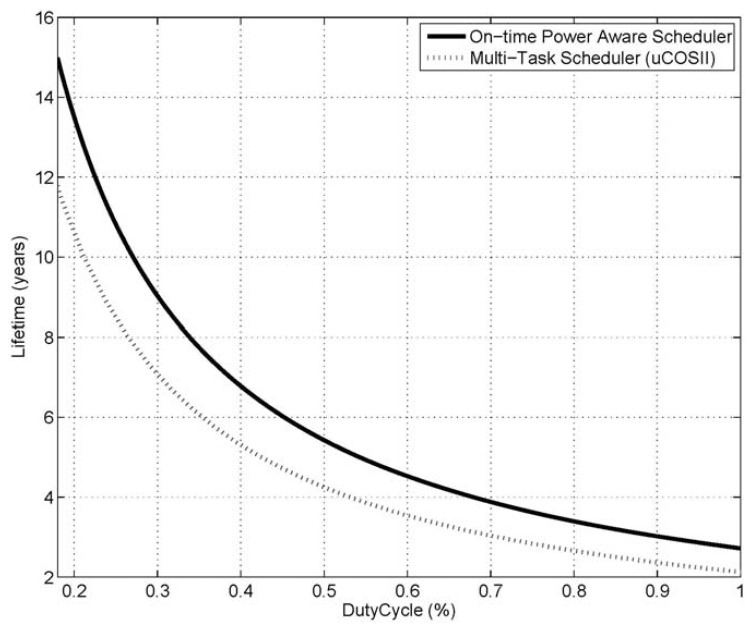
Comparison of lifetimes of the two systems.

Using the lifetime estimation method given above Equations (1) and (2), the power consumption of the hardware devices shown in Figure 9 was modelled using the Mathworks Matlab simulation tool. Three inputs were given to the lifetime simulation model: (1) the amounts of power consumed in the stepwise data transmission processing, (2) the power consumption and saving ratios of the power controllable hardware components, and (3) the duty-cycle, which represents the ratio of the processing time of data transmission/reception. In this simulation, a 3,000 mAh battery was assumed, and duty-cycle values ranging from 0.2% to 1% were used to determine the work times of the power modes. Figure 13 shows the resulting comparison of the lifetimes of the two systems.

The results show that the lifetime of the on-time power-aware scheduler is approximately 30% longer than that of the μCOSII-based system. This improvement was the straightforward result of the intelligent power adjustment that was made based on the prediction of executions in the system's active mode. In the μCOSII-based system, *wait_event* cannot be used, and the *sleep_with_timer* mode can hardly be used because it does not support such predictive scheduling. In addition, the μCOSII-based system must circulate non-optimized power when the system is active, which leads to quite large differences in the amounts of power that are consumed in the active and inactive modes of the two systems. Another reason for the power saving is that the scheduler can operate with a single stack memory because there is no pre-emption, even in the scheduling of multiple tasks. Thus, the scheduler can reduce the scheduling latency and the size of the volatile memory, which leads to faster sleep and wake-up operations. To compare the scheduling latencies of the developed system and the μCOSII-based system, an application that performs the acquisition of data from a sensor and the transmission of data to an external device was executed 20 times. As shown in the results in Table 3, the new system shows, at worst, a scheduling latency reduction of about 5.3%.

Table 4 presents the MCU utilizations and code sizes of the two systems when the same application was launched. The difference in the codeSS sizes is mainly due to the size of the kernels. The MCU utilization factors represent when the systems are active. In cases in which the developed scheduler was used, the MCU utilization was reduced to 2.32%. This result is attributed to the fact that the developed system is smaller and does not need any synchronization primitives because it does not perform pre-emption.

## 5. Conclusions

In order to achieve low-power and reliable on-time periodic operations of a medical sensor device, management of the power mode of the system based on the prediction of the timing behaviours of tasks is most important. This paper has reported on the development of a new on-time, power-aware scheduler based on dual priority scheduling [18] and the TMO's BCC scheme [16]. The scheduler supports three key features. The first is that periodic tasks are serialized without pre-emption or delay. This eliminates the synchronization overheads between tasks and makes it possible to have predictable power management. The second feature is the postponement of a sporadic task for the on-time scheduling of an important periodic task when an overlap between their execution is expected in the near future. The third feature is the support of the optimized power mode, which is activated according to each possible scheduling scenario. For purposes of commercialization following field tests, the scheduler was implemented in a battery-powered electrical impulse generator that was developed as an SoC to prevent sudden cardiac death due to ventricular fibrillation and ventricular tachycardia. The experimental and analytical results show that the developed scheduler can increase the system's lifetime by up to 30%, as compared to a commercial RTOS-based system, when the duty-cycle is less than 1% in a WBAN node.

The developed system will be deployed in complex sensor systems that perform acquisitions of live body signals and audio/video and environmental information on a textile-based wearable computing platform. To deploy the new scheduler in a sensor-based system, the last remaining aspect to be clarified involves classifying the power mode of a new system case by case, in order to optimize the power consumption.

## Figures and Tables

**Table 1.** PHY characteristics of the hardware MAC.

Frequency Band	402–405 MHz
Channel/BW	10 (300 kHz/Ch)
Modulation	DBPSK/DQPSK
Data Rate	300 kbps
FEC	BCH
Pulse Shape Filter	Gaussian Filter
Power Consumption	Avg. 12 mA
Hardwired Low-MAC	Auto-CRC/FEC, Auto-ACK MAC Frame Handling
RF Wakeup	2.45 GHz (OOK, Avg. 235 nA)

**Table 2.** Power modes and measured current in each mode.

**Power Mode**	**Avg. Current (μA)**	**Description**
PM0	0.235	Sleep with radio mode
PM1	820	Sleep with timer mode
PM2	4,023	Waiting for an event: 32.768 kHz MCU Enables half memory one or more standby devices
PM3	9,944	Processing data: 6 MHz MCU Enables all memory timer, ADC blocks, standby transceiver
PM4	17,402	Transmitting frames: full-speed MCU Enables all memory, transmitter, and required IO blocks
PM5	18,532	Receiving Frames: full-speed MCU Enables all memory, receiver requires IO blocks

**Table 3.** Comparison of scheduling latencies.

		**Multi-tasking Scheduler (μCOSII)**	**On-time Power-aware Scheduler**
Context Switching Time (μs)		35	21
Scheduling Overhead (μs)	Worst	189	170
	Best	170	166
	Average	175	168

**Table 4.** Comparison of system resources required.

	**Multi-tasking Scheduler (μCOSII)**	**On-time Power-aware Scheduler**
**MCU Utilization**	5.07%	2.32%
**Code Size in Bytes (Data/Program, μs)**	5,065/42,155	1,958/33,679

## References

[1] Lo B., Yang G. Body Sensor Networks—Research Challenges and Opportunities.

[2] Gust H.B., Warren M.S., Margaret A.H., Ian G.C., Iain C.M., Luc J., Dominic T., Robert E.P., David J.W., Derek T.C. (2010). An Entirely subcutaneous implantable cardioverter-defibrillator. N. Engl. J. Med..

[3] Chen M., Gonzalez S., Vasilakos A., Cao H. (2010). Body area networks: A survey. Mobile Networks Appl..

[b4-sensors-13-00375] 4.IEEE 802.15.6 TG6. Available online: http://www.ieee802.org/15/pub/TG6.html (accessed on 29 February 2012).

[5] Davenport D.M., Ross F.J. Wearable and Implantable Body Sensor Networks for Ambulatory Patient Monitoring.

[6] Bohorquez J.D., Chandrakasan A. A 350W CMOS MSK Transmitter and 400W OOK Super-Regenerative Receiver for Medical Implant Communications.

[7] Higgins H. Body Implant Communication—Is it a Reality.

[8] Miller M.J., Vaidya N.H. (2004). Minimizing energy consumption in sensor networks using a wakeup radio. Wireless Commun. Network. Conf..

[9] Ameen M.A., Ullah N., Kwak K. Design and Analysis of a Mac Protocol for Wireless Body Area Network Using Wakeup-Radio.

[10] Shankar V., Schwiebert L. Energy-efficient Protocols for Wireless Communication in Biosensor Networks.

[11] Wang X., Vasilakos A.V., Chen M., Liu Y., Kwon T.T. (2012). A survey of green mobile networks: Opportunities and challenges. Mobile Networks Appl..

[12] Chilamkurti N., Zeadally S., Vasilakos A., Sharma V. (2009). Cross-layer support for energy efficient routing in wireless sensor networks. J. Sensors.

[13] TinyOS http://www.tinyos.net/.

[14] Shah B., James C., Hui D., Jing D., Jeff R., Anmol S., Brian S., Charles G., Adam T., Richard H. (2005). MANTIS OS: An embedded multithreaded operating system for wireless micro sensor platforms. ACM Kluwer Mobile Networks Appl. (MONET).

[15] Kim K.H., Kopetz H. A Real-Time Object Model RTO.k and an Experimental Investigation of Its Potentials.

[16] Kim J.G., Kim M.H., Heu S. Architectures and Functions of the TMO Kernels for Ubiquitous & Embedded Real-Time Distributed Computing.

[17] Davis R., Wellings A. Dual Priority Scheduling.

[18] Moncusi M.A., Arenas A., Labarta J. (2001). Improving energy saving in hard real time systems via a modified dual priority scheduling. SIGARCH Comput. Archit..

[19] Kim H., Kim J.G. (2008). An Efficient Task Serializer for Hard Real-time TMO Systems.

[20] Sukor M., Ariffin S. Performance Study of Wireless Body Area Network in Medical Environment.

[21] Richard A., Comroe D.J., Costello (1984). ARQ schemes for data transmission in mobile radio systems. IEEE J. Selective Areas Commun..

[22] μCOSII http://micrium.com/page/home.

[23] Mainwaring A., Polastre J., Szewczyk R., Culler D., Anderson J. Wireless Sensor Networks for Habitat Monitoring.

